# Micronuclei Formation upon Radioiodine Therapy for Well-Differentiated Thyroid Cancer: The Influence of DNA Repair Genes Variants

**DOI:** 10.3390/genes11091083

**Published:** 2020-09-17

**Authors:** Luís S. Santos, Octávia M. Gil, Susana N. Silva, Bruno C. Gomes, Teresa C. Ferreira, Edward Limbert, José Rueff

**Affiliations:** 1 Centre for Toxicogenomics and Human Health (ToxOmics), Genetics, Oncology and Human Toxicology, NOVA Medical School; Faculdade de Ciências Médicas, Universidade Nova de Lisboa, 1169-056 Lisboa, Portugal; lsilvasantos@gmail.com (L.S.S.); bruno.gomes@nms.unl.pt (B.C.G.); jose.rueff@nms.unl.pt (J.R.); 2 Institute of Health Sciences (ICS), Center for Interdisciplinary Research in Health (CIIS), Universidade Católica Portuguesa, 3504-505 Viseu, Portugal; 3 Centro de Ciências e Tecnologias Nucleares, Instituto Superior Técnico, Universidade de Lisboa, 2695-066 Bobadela, Loures, Portugal; ogil@ctn.tecnico.ulisboa.pt; 4 Serviço de Medicina Nuclear, Instituto Português de Oncologia de Lisboa (IPOLFG), 1099-023 Lisboa, Portugal; teresa.ferreira_medical@yahoo.com; 5 Serviço de Endocrinologia, Instituto Português de Oncologia de Lisboa (IPOLFG), 1099-023 Lisboa, Portugal; elimbert@ipolisboa.min-saude.pt

**Keywords:** thyroid cancer, Iodine-131, chromosome-defective micronuclei, DNA repair, micronucleus assay, single nucleotide polymorphism, pharmacogenomic variants, pharmacogenetics, precision medicine

## Abstract

Radioiodine therapy with ^131^I remains the mainstay of standard treatment for well-differentiated thyroid cancer (DTC). Prognosis is good but concern exists that ^131^I-emitted ionizing radiation may induce double-strand breaks in extra-thyroidal tissues, increasing the risk of secondary malignancies. We, therefore, sought to evaluate the induction and 2-year persistence of micronuclei (MN) in lymphocytes from 26 ^131^I-treated DTC patients and the potential impact of nine homologous recombination (HR), non-homologous end-joining (NHEJ), and mismatch repair (MMR) polymorphisms on MN levels. MN frequency was determined by the cytokinesis-blocked micronucleus assay while genotyping was performed through pre-designed TaqMan^®^ Assays or conventional PCR-restriction fragment length polymorphism (RFLP). MN levels increased significantly one month after therapy and remained persistently higher than baseline for 2 years. A marked reduction in lymphocyte proliferation capacity was also apparent 2 years after therapy. *MLH1* rs1799977 was associated with MN frequency (absolute or net variation) one month after therapy, in two independent groups. Significant associations were also observed for *MSH3* rs26279, *MSH4* rs5745325, *NBN* rs1805794, and tumor histotype. Overall, our results suggest that ^131^I therapy may pose a long-term challenge to cells other than thyrocytes and that the individual genetic profile may influence ^131^I sensitivity, hence its risk-benefit ratio. Further studies are warranted to confirm the potential utility of these single nucleotide polymorphisms (SNPs) as radiogenomic biomarkers in the personalization of radioiodine therapy.

## 1. Introduction

Thyroid cancer (TC) is the most common endocrine malignancy, accounting for approximately 2.1% of cancers diagnosed all over the world. TC incidence is about two to four times higher in women than in men and is one of the most common malignancies in adolescent and young adults (ages 15–39 years), with the median age at diagnosis being lower than that for most other types of cancer [1,2,3]. TC incidence has been steadily increasing, over the last three decades [1], most likely because of “surveillance bias” and overdiagnosis resulting from increased detection of small stationary lesions of limited clinical relevance. A true rise in the number of TC cases (e.g., due to increasing exposure to ionizing radiation (IR) from medical sources) is, however, also possible [2,3,4].

Papillary (PTC) and follicular (FTC) thyroid carcinoma represent 85–90% and 5–10% of TC cases, respectively. These tumor histotypes retain their morphologic features, being often referred to as differentiated thyroid carcinoma (DTC) [3,4]. The best-established modifiable risk factor for DTC is IR exposure during childhood and adolescence (radioiodines including ^131^I, X-radiation, γ-radiation) [2,3,4,5] and the standard treatment consists of surgical resection (total or near-total thyroidectomy) accompanied by post-thyroidectomy radioiodine (RAI) therapy and TSH suppression [3,4]. The majority of DTC cases is indolent in nature, iodine-avid, and responds favorably to standard therapy. Overall prognosis is thus generally good, translating into high long-term survival and low disease-specific mortality [4].

The widespread use of RAI therapy in the management of DTC relies on the ability of ^131^I to be preferentially taken up and concentrated in normal or neoplastic thyroid follicular cells, taking advantage of these cells’ specialized mechanism for iodide uptake and accumulation [3,6,7]. Thyrocyte-accumulated ^131^I undergoes [β and γ] decay and releases high-energy electrons that inflict devastating DNA damage locally. Thyroid cell death through radiation cytotoxicity ensues, allowing for the ablation of remnant normal thyroid tissue and the eradication of any residual tumor foci [3,6]. Unfortunately, since other tissues may also concentrate ^131^I, its DNA damaging effects may not be limited to the thyroid gland, increasing the risk of RAI-associated secondary malignancies such as soft tissue tumors, colorectal cancer, salivary tumors, and leukemia [3,7]. Since the rising incidence of TC is mostly driven by increased detection of stationary subclinical lesions, concern exists that DTC overdiagnosis may result in potentially harmful overtreatment [2]. Indeed, if we consider the indolent behavior of the disease, its long-term survival rate, and its mean age of diagnosis, such therapy-related morbidity may not be justified, as most patients will have many years to experience its negative effects [2]. The revised American Thyroid Association (ATA) clinical practice guidelines for the management of DTC [8] reflect such concern for the first time, recommending a more cautious diagnosis and treatment approach in order to reduce RAI use (hence, radiation exposure) particularly in younger ages. This includes, for example, more stringent criteria for diagnosis upon nodule detection, molecular-based risk stratification for improved treatment decisions, personalized disease management and long-term surveillance strategies and, most importantly, use of lower RAI doses (30–50 mCi) in patients with low-risk DTC [2,8,9].

The most relevant types of DNA damage inflicted upon IR exposure are double-strand breaks (DSBs). Such lesions are predominantly processed by DNA repair enzymes of the homologous recombination (HR) and non-homologous end-joining (NHEJ) repair pathways, despite mismatch repair (MMR) pathway enzymes have also been implicated [10,11]. The activity of such DNA repair enzymes determines the capacity of cells to repair DSBs which, in turn, influences their sensitivity to IR. Lower DNA repair capacity, therefore, increases the extent of IR-induced DNA damage, increasing both the likelihood of cell death through IR-induced cytotoxicity and the likelihood of malignant transformation upon IR exposure [12,13].

Single nucleotide polymorphisms (SNPs) in DNA repair enzymes across these three pathways have been identified and some have been demonstrated to affect the DNA repair capacity [14,15]. Such DNA repair SNPs may therefore modulate sensitivity to IR and many have indeed been associated with TC or, more specifically, DTC susceptibility (for which IR exposure is the best-established risk factor) [16,17,18,19,20,21]. It is likely that such functional DNA repair SNPs, through interference with the extent of IR-induced DSBs on thyrocytes, could influence the cytotoxic potential of RAI therapy, hence its efficacy on DTC treatment. Likewise, through a similar effect on other cells that take up and concentrate ^131^I, such SNPs could also modify the risk of secondary malignancies, hence the safety of RAI therapy. Identifying these variants is, therefore, an important challenge with clinical relevance. However, to our knowledge, the issue has not been addressed in prior studies.

We have previously demonstrated that therapy with 70 mCi ^131^I in DTC patients is consistently associated with increased DNA damage levels in peripheral lymphocytes [22,23]. With this study, we aimed to confirm, through the use of the cytokinesis-blocked micronucleus (CBMN) assay, our prior findings in a new group of DTC patients submitted to RAI therapy with 100 mCi. Further, we sought to extend our analysis at 24 months after ^131^I administration so that the long-term persistence of ^131^I-induced DNA damage could be better characterized. Finally, the potential influence of HR, NHEJ, and MMR polymorphisms on the micronuclei (MN) frequency in RAI-treated DTC patients was also investigated.

Understanding the role of repair SNPs on the extent and persistence of ^131^I-induced DNA damage will contribute to the identification of genetic biomarkers that influence the individual response to ^131^I-based RAI therapy and thus modulate the risk-benefit ratio of RAI therapy in DTC patients. Such efforts may provide the basis for improved, personalized, therapeutic decisions in the context of DTC therapy, with impact on disease prognosis and patient safety.

## 2. Materials and Methods

### 2.1. Study Population

Twenty-six DTC patients proposed for radioiodine therapy at the Department of Nuclear Medicine of the Portuguese Oncology Institute of Lisbon (Portugal) were selected according to criteria published elsewhere [22]. All participants were treated according to current practice, consisting of total thyroidectomy followed by oral administration of ^131^I, 70 mCi (15 patients) or 100 mCi (11 patients), to ablate thyroid remnant cells. Patients were followed for two years unless they had to be submitted to further treatment. In such cases, patients were no longer elective for cytogenetic analysis and had to be excluded from further analysis. A mixed cross-sectional and longitudinal study design was used, respectively, for comparisons among genotypes or dose groups at each time point and across different time points. In the latter case, pre-treatment values allowed each patient to serve as his own control.

To characterize the study population and account for potential confounding factors, all participants were interviewed and completed a detailed questionnaire covering standard demographic characteristics, personal and family medical history, lifestyle habits, and prior IR exposure. For the purpose of smoking status, former smokers who had quit smoking at least 2 years prior to diagnosis were considered as non-smokers. Clinical and pathological examination was also performed.

Peripheral blood samples were collected from each patient into both 10 mL heparinized tubes (for cytogenetic analysis) and citrated tubes (for genotype analysis). For cytogenetic analysis, blood samples were drawn (1) prior to ^131^I administration as well as 1, 6, and 24 months after therapy in patients submitted to a 70 mCi dose and (2) prior to ^131^I administration as well as 1 and 3 months afterward in patients submitted to a 100 mCi dose. For genotype analysis, blood samples were stored at −80 °C until further use.

All subjects gave their informed consent for inclusion before they participated in the study. The study was conducted in accordance with the Declaration of Helsinki, and the protocol was approved by the Ethics Committee of Instituto Português de Oncologia Francisco Gentil (GIC/357) and by the Ethics Committee of Faculdade Ciências Médicas (CE-5/2008).

### 2.2. Genotype Analysis

Genomic DNA was isolated from blood samples using the commercially available QIAamp^®^ DNA mini kit (QIAamp^®^ DNA mini kit; Qiagen GmbH, Hilden, Germany), according to the manufacturer’s recommendations. The fluorimetric Quant-iT™ Picogreen^®^ dsDNA Assay Kit (Invitrogen, Waltham, MA, USA) was used to quantify and ensure uniformity in DNA concentration (2.5 ng/µL). DNA samples were kept at −20 °C until further use.

SNPs were selected from those already analyzed by our team in a cohort of 106 DTC patients, according to selection criteria published elsewhere [18,19,20,21]. Due to sample size limitations, only SNPs presenting a minor allele frequency (MAF) > 0.15 in the original pool of patients were considered. *MLH3* rs175080 was excluded *a posteriori* for insufficient genotype frequency (*n* ≤ 1) in at least one of the ^131^I dose groups (Table S1). Overall, a total of 9 DNA repair SNPs across 3 DNA repair pathways (HR, NHEJ, and MMR) were considered for further analysis (Table 1).

Genotyping was performed mostly by real-time polymerase chain reaction (RT-PCR): amplification and allelic discrimination were carried out on a 96-well ABI 7300 Real-Time PCR system thermal cycler (Applied Biosystems; Thermo Fisher Scientific, Inc., Waltham, MA, USA), following the manufacturer’s instructions, with the use of the commercially available TaqMan^®^ SNP Genotyping Assays (Applied Biosystems) identified in Table 1. For *XRCC3* rs861539 (HR pathway), genotyping was performed by conventional PCR-restriction fragment length polymorphism (RFLP) techniques. Primer sequences, PCR, and digestion conditions as well as expected electrophoretic patterns have been described [19]. To confirm genotyping and ensure accurate results, inconclusive samples were reanalyzed and genotyping was repeated in 10–15% of randomly chosen samples, with 100% concordance.

### 2.3. Cytogenetic Analysis

The cytokinesis-block micronucleus assay (CBMN) was used to analyze DNA damage and conducted according to standard methods. The methodology was performed and published as described previously [22,23,24]. The frequency of binucleated cells carrying micronuclei (BNMN), defined as the number of cells with MN per 1000 binucleated lymphocytes, is expressed as a count per thousand (‰). The Cytokinesis-Block Proliferation Index (CBPI) was determined according to the formula CBPI = [MI + 2MII + 3(MIII + MIV)]/N, where MI-MIV correspond to the number of human lymphocytes with one to four nuclei, respectively, and N is the total number of cells analyzed.

### 2.4. Statistical Analysis

All analyses were done with SPSS 22.0 (IBM SPSS Statistics for Windows, version 22.0, IBM Corp, Armonk, NY, USA) except for deviation of genotype distributions from Hardy–Weinberg equilibrium (HWE) and linkage disequilibrium (LD) analysis between SNPs on the same chromosome, which were performed with SNPstats [25].

Categorical variables, presented as frequencies and percentages, were compared between dose groups and with the original cohort of DTC patients by the Pearson’s Chi-square (χ^2^) test or the two-sided Fisher’s exact test whenever 2 × 2 contingency tables were possible. For continuous variables (BNMN frequency, CBPI, and their net variation from baseline), presented as mean ± standard deviation, the normality and homogeneity of variances were evaluated by the Shapiro-Wilk and Levene tests, respectively. Longitudinal comparisons were performed by the paired sample *t* test (whenever a normal distribution could not be excluded) or the Wilcoxon signed-rank test (remaining cases) while the parametric Student t test (normal distributions) or the nonparametric Mann-Whitney U test (non-normal distributions) for independent samples were used for cross-sectional comparisons between the two ^131^I dose groups and between different gender, age class, smoking status, histological type of tumor, and genotype categories.

Variable transformation was considered, when practically useful: DTC patients were dichotomized according to age, with the cut-off point being defined as the median age of all patients included (54 years). Due to limited sample size (hence, low frequency of homozygous variant genotypes), a dominant model of inheritance was assumed for all SNPs. Moreover, the net variation in BNMN frequency (i.e., therapy-induced BNMN) was calculated by subtracting the background (pre-treatment) BNMN frequency from the corresponding post-treatment values.

This is an exploratory ‘proof of concept’ study, not a conclusive final one. As such, the Bonferroni adjustment was deemed as not necessary as it is too conservative. Furthermore, the complement of the false-negative rate β to compute the power of a test (1-β) was not taken into account at this stage since larger studies are needed to change this preliminary study into a confirmatory one. Statistical significance was set at *p* < 0.05.

## 3. Results

### 3.1. Characteristics of the Study Population

A general description of the study population is presented in Table 2. The age of DTC patients submitted to ^131^I therapy ranged from 32 to 73 years, with a mean of 52.54 ± 11.62 years. As expected, female patients (88.5%, *n* = 23) greatly outnumbered male patients (11.5%, *n* = 3) and papillary carcinoma cases (PTC, 69.2%, *n* = 18) were also more frequent than follicular ones (FTC, 30.8%, *n* = 8), in agreement with gender and histotype distributions commonly reported for DTC [1,2,4]. Overall, 15.4% (*n* = 4) of patients were smokers. No significant differences in patient age, gender, histological type of tumor, and smoking status were observed between groups submitted to different ^131^I doses (Table 2) nor between any of these groups (separated or together) and our original DTC population [18].

### 3.2. Cytogenetic Data

**Figure 1:**
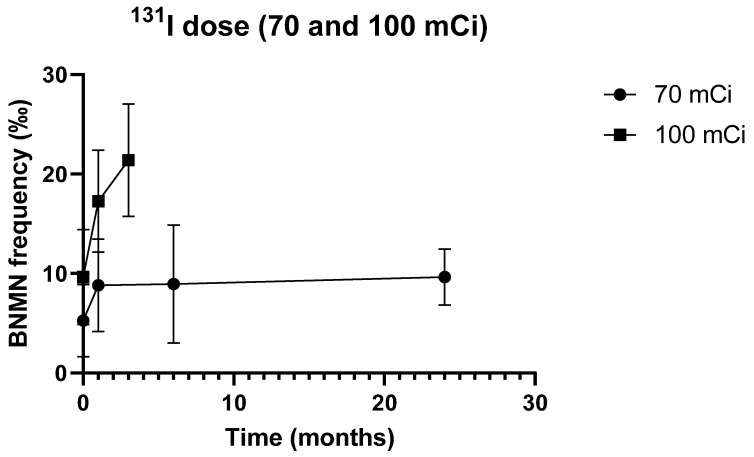
Binucleated cells carrying micronuclei (BNMN) frequency (‰, mean ± S.D.) in DTC patients before and after (1, 3/6, and 24 months) therapy with different doses of ^131^I (70 and 100 mCi).

The frequency of BNMN (mean ± S.D.) in the 26 DTC patients submitted to ^131^I therapy and included in this study is illustrated in Figure 1 and summarized in Table S2. Pre-treatment and post-treatment values are presented, stratified by dose group.

The results from the 70 mCi dose group until 6 months after ^131^I administration have been published before [22]. As it was not possible to collect genotyping data on 4 of the original 19 patients, these patients were excluded and the data were re-analyzed. Longitudinal results in this dose group are, nevertheless, similar to those originally reported [22]: as evident from Figure 1, BNMN frequency in these patients increases significantly 1 month after ^131^I therapy (from 5.27 ± 3.63‰ to 8.80 ± 4.65‰, *p* = 0.039) and stabilizes at 6 months after ^131^I therapy (8.93 ± 5.92‰, *p* = 0.944 vs. 1 month after therapy), remaining persistently higher than before treatment (*p* = 0.041).

To investigate the long-term persistence of such therapy-induced damage, the study of these patients at 2 years after therapy was extended (Table S2 and Figure 1). Cytogenetic data at such time point was available for 11 patients only. The frequency of BNMN remained stable (9.64 ± 2.80‰, similar to values at 1 and 6 months, *p* = 0.460 and *p* = 0.328, respectively) and persistently higher than baseline (*p* = 0.005).

To confirm these findings and check for a possible dose effect, the study was replicated in an independent group of patients administered with 100 mCi. As expected, BNMN frequency was significantly higher in the 100 mCi group than in the 70 mCi group, irrespective of the time point (Table S2 and Figure 1), suggesting a dose-effect association (hence, a cause-effect relation) between iodine dose and BNMN levels. Apart from this quantitative difference, the effect of either dose on BNMN frequency was qualitatively similar, BNMN in the 100 mCi group increasing significantly 1 month after therapy (from 9.64 ± 4.78‰ to 17.27 ± 5.14‰, *p* = 0.011) and remaining persistently higher than baseline at 3 months (21.40 ± 5.66‰, *p* < 0.001 and *p* = 0.054 compared to pre-treatment and 1 month post-treatment values, respectively) (Table S2).

Moreover, of notice, the BNMN increment (net balance) after ^131^I therapy was more pronounced in the 100 mCi group than in the 70 mCi group, despite the difference was not significant (*p* > 0.05).

**Figure 2:**
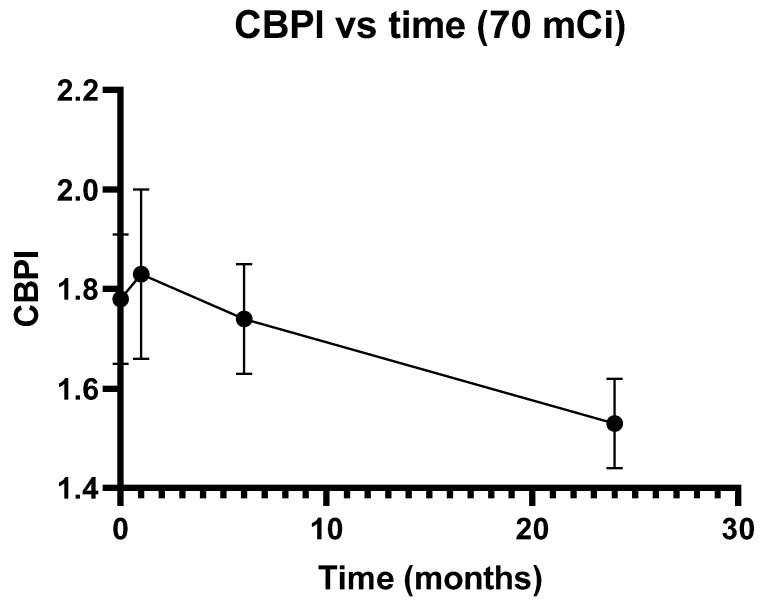
Cytokinesis-Block Proliferation Index (CBPI) (mean ± S.D.) in DTC patients before and after (1, 6, and 24 months) therapy with ^131^I (70 mCi).

Finally, the CBPI (mean ± S.D.) was also determined for the 15 DTC patients submitted to therapy with 70 mCi ^131^I. As depicted in Figure 2, this index, which indicates the proliferation capacity of lymphocytes and may be used to calculate cytotoxicity [26], did not change appreciably at 1 and 6 months after ^131^I administration but was markedly reduced at 24 months after therapy (from 1.78 ± 0.13 to 1.53 ± 0.09, *p* = 0.001).

### 3.3. Characteristics of the Study Population and Cytogenetic Data

**Figure 3:**
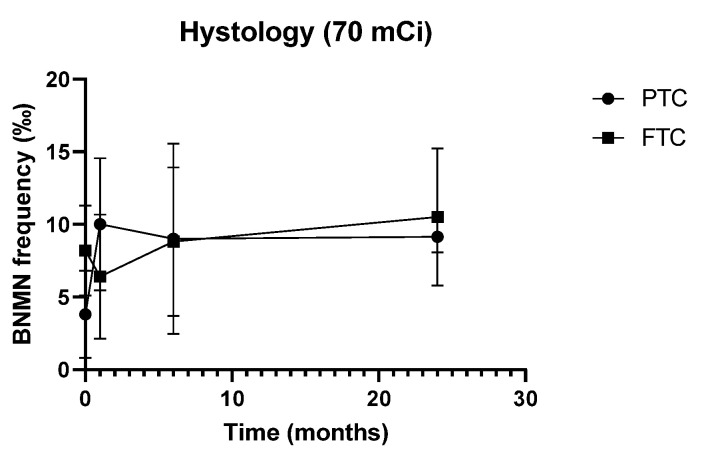
BNMN frequency (‰, mean ± S.D.) in DTC patients before and after (1, 6, and 24 months) therapy with 70 mCi ^131^I, according to tumor histotype (papillary thyroid carcinoma (PTC) and follicular thyroid carcinoma (FTC)).

The potential influence of the demographic, lifestyle, and clinical characteristics of the study population on cytogenetic data was also evaluated. As depicted in Figure 3, in patients treated with 70 mCi, histology interfered with both pre-treatment BNMN levels and its net balance 1 month after ^131^I therapy (Figure 3): basal BNMN frequency was significantly higher in FTC than in PTC patients (8.20 ± 3.11‰ vs. 3.80 ± 3.01‰, *p* = 0.020) but, 1 month after therapy, increased only in PTC patients, resulting in a significantly different net balance between the two histotypes (+6.20 ± 5.05‰ in PTC vs. −1.80 ± 3.96‰ in FTC, *p* = 0.009). Such effect was not observed in 100 mCi-treated patients nor when both dose groups were considered together. Likewise, no significant effect of gender, age, or smoking habits on BNMN levels or its net balance was detected, irrespective of the time point or dose group. Furthermore, except maybe for gender, no significant effect on CBPI was observed for any of these variables in the 70 mCi dose group. Baseline CBPI values were borderline higher in female compared to male patients (*p* = 0.045) but such finding should not be overvalued as only one male patient was included in this dose group.

### 3.4. Distribution of DNA Repair SNPs in the Study Population

Table 3 reports the allele frequency and genotype distribution of 9 DNA repair SNPs among our sample of ^131^I-treated patients. Genotype distributions were consistent with HWE in either dose group or their combination (*p* > 0.05) and, except for *MSH3* rs26279, did not differ significantly from those described in our previously studied DTC population (^c^). For *MSH3* rs26279, non-uniform distribution was observed, with the common allele being overrepresented in the study sample compared to the original population (*p* = 0.048, in the dominant model, Table S1). Moreover, importantly, no significant differences in genotype distributions were detected between dose groups, for any of the SNPs, irrespective of the model of inheritance assumed (Table 3). No relevant linkage association was observed between any of the SNPs.

### 3.5. DNA Repair SNPs and Cytogenetic Data

**Figure 4:**
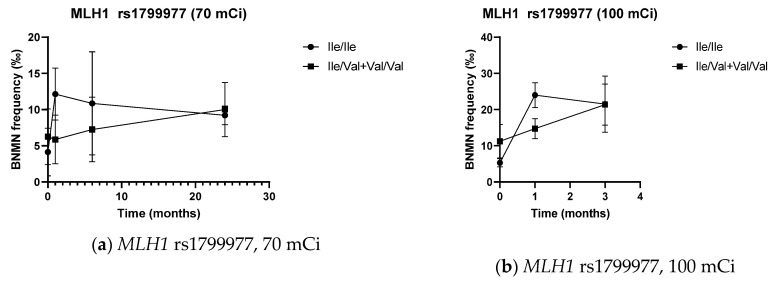

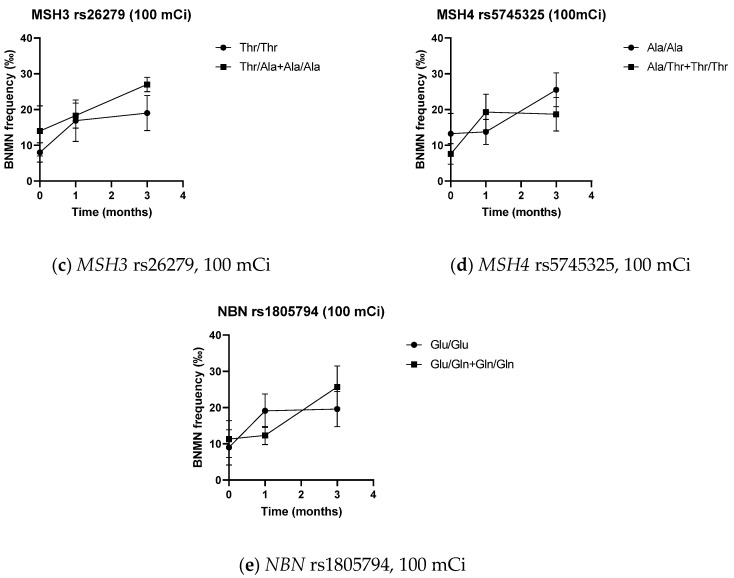
BNMN frequency (‰, mean ± S.D.) in DTC patients before and after (1, 3/6, and 24 months) therapy with ^131^I, according to genotype and ^131^I dose group: (**a**) *MLH1* rs1799977, 70 mCi; (**b**) *MLH1* rs1799977, 100 mCi; (**c**) *MSH3* rs26279, 100 mCi; (**d**) *MSH4* rs5745325, 100 mCi; (**e**) *NBN* rs1805794, 100 mCi.

The influence of DNA repair SNPs on BNMN frequencies and the corresponding variation from pre-treatment values is shown in Figure 4, Table 4, Table 5 and Tables S3–S5.

Prior to ^131^I administration, BNMN frequency was higher in patients carrying the *MLH1* rs1799977 variant allele than in those homozygous for the common allele, with the difference being significant in the 100 mCi dose group (*p* = 0.012) and in the pool of both groups (*p* = 0.019).

One month after ^131^I administration, *MLH1* rs1799977 variant allele carriers always presented significantly lower BNMN levels than patients homozygous for the common allele, either when considering absolute values (*p* = 0.004, *p* = 0.012 and *p* = 0.034 in the 70 mCi, 100 mCi, and in the pool of both groups, respectively) or the net variation from baseline (*p* = 0.002, *p* = 0.001 and *p* < 0.001 in the 70 mCi, 100 mCi and in the pool of both groups, respectively). BNMN frequency one month after therapy was also significantly lower in carriers of the variant allele for *NBN* rs1805794 (*p* = 0.043 in the 100 mCi group and *p* = 0.017 in the pool of both groups), with the difference in net BNMN values almost being significant (*p* = 0.099 in the 100 mCi dose group and *p* = 0.058 in the pool of both groups). Further, carriers of at least one *MSH4* rs5745325 variant allele exhibited higher levels of ^131^I-induced BNMN than patients homozygous for the common allele (*p* = 0.018 in the 100 mCi group, *p* = 0.043 in the combination of both groups), with the difference in absolute BNMN frequencies being significant in the pooled analysis of both groups (*p* = 0.039) and almost significant in the 100 mCi group (*p* = 0.084).

Three months after therapy, significantly higher BNMN frequencies were found in patients from the 100 mCi group carrying the *MSH3* rs26279 variant allele (*p* = 0.030).

No other significant difference in either absolute or therapy-induced BNMN frequencies was found between the different genotypes of the DNA repair SNPs, at any time point. Likewise, no influence of genotype in CBPI, either absolute or relative to baseline values, was detected for any of the DNA repair SNPs considered in this study, at any time point (Table S6).

## 4. Discussion

We have previously demonstrated a significant increase in BNMN frequency in peripheral lymphocytes from 19 DTC patients treated with 70 mCi ^131^I [22]. In the present exploratory study, in order to confirm these findings, to evaluate the long-term persistence of such ^131^I-induced DNA damage and to determine whether it may be influenced by DNA repair SNPs, we extended our analysis at 2 years after ^131^I administration in this group of patients, included a new group of patients submitted to RAI therapy with 100 mCi and profiled 9 DNA repair SNPs in patients from both groups.

In line with our previously reported results, we observed, in the 100 mCi dose group, a significant and persistent increase in BNMN frequency after ^131^I therapy, with mean levels being always higher than in the 70 mCi group, irrespective of the time point considered. Replication across two independent sets of patients and observation of a dose effect strongly suggests a causal relation between RAI therapy and systemic chromosomal damage in lymphocytes, as assessed by the MNCB assay. Such correlation has been repeatedly demonstrated (both in thyroid patients following RAI therapy [27,28,29,30,31,32] and in other settings where exposure to low levels of low-LET (linear energy transfer) ionizing radiation occurs [28,33]) and is expected since ^131^I may be taken up by extra-thyroidal cells [7] and emit β- and γ-radiation capable of inducing dose-dependent chromosomal damage detectable by cytogenetic analysis (e.g., micronuclei) [27,28,32]. The ability of ^131^I to induce cytogenetic damage in peripheral lymphocytes in a dose-dependent manner is, in fact, clear and well-established, allowing BNMN frequency to be used as a valid, highly sensitive, and specific biomarker of effect for biological dosimetry of RAI therapy and, hence, to predict its associated genotoxic risk in dividing mammalian cells [27,28,32,34,35].

A less clear picture exists, however, concerning the long-term persistence (kinetics of the recovery) of such IR-induced cytogenetic damage. Our results from the 70 mCi dose group suggest that ^131^I-induced damage in peripheral lymphocytes persists for at least 2 years. Despite negative results have also been published [36,37], our results are in line with most prior follow-up studies on RAI therapy or other low-dose IR exposures (e.g., for diagnostic purposes) [28,29,38,39,40,41]. Considering the half-life of ^131^I (ranging from 1 to 8 days in thyroidectomized and non-thyroidectomized TC patients, respectively) [28] and of circulating lymphocytes (about 3 years) [28,38], such repeated demonstration of persistent cytogenetic damage is somehow surprising and challenge the widely held views about the mechanisms of IR-induced DNA damage. Possible explanations for the long-term genomic instability of lymphocytes from ^131^I-exposed subjects include the introduction, upon irradiation, of DNA damage and cytogenetic alterations (1) in a subset of long-lived naïve T lymphocytes, quiescent cells that survive for prolonged periods of time in a resting stage, retaining the initially inflicted DNA damage and expressing it as micronuclei when stimulated to proliferate in the CBMN assay [38,42,43], (2) in hematopoietic stem and progenitor cells that, through clonal expansion, may give rise to mature T lymphocytes with stable and unstable aberrations, perpetuating genomic instability in time (transgenerational effect) [38,42,43], and (3) in non-irradiated lymphocytes (a delayed non-targeted effect), as a result of the long-term production and plasma secretion of soluble clastogenic factors by irradiated cells (oxidative stress by-products such as ROS (reactive oxygen species) and inflammatory cytokines such as TNF-α) that may further extend IR-induced cytogenetic damage in time (“bystander effect”) [44]. The two latter explanations are generally favored, as a large number of studies exist demonstrating either the high frequency of gene mutations and chromosomal aberrations in the progeny of irradiated cells or the production and plasma release of factors with clastogenic activity by irradiated cells (including one on ^131^I-treated patients) [37]. Overall, current evidence [44,45,46,47] supports the notion that a potent long-term inflammatory-type response develops upon IR exposure, irradiated cells producing danger signals (oxidative stress by-products and inflammatory cytokines) capable of exerting an array of persistent bystander effects in non-irradiated cells (altered levels of damage-inducible and stress-related proteins), leading to delayed genomic instability (chromosomal aberrations, sister chromatid exchanges, micronuclei formation/induction or mutations), hence, predisposing to malignancy (altered proliferation or transformation). Such long-term inflammatory-type response could also be responsible for the marked reduction in CBPI that we observed at 24 months after ^131^I therapy.

In this study, complying with current recommendations, we also investigated the role of potential confounding factors on BNMN frequency. As reviewed elsewhere [48,49,50] and demonstrated through meta-analysis in the International Human MicroNucleus (HUMN) Project [51], age and gender are well-established factors, with increasing age and female gender being consistently associated with higher BNMN levels in peripheral blood lymphocytes. The influence of age has been demonstrated, in particular, in ^131^I-treated patients [28,31]. Data on the potential role of smoking status on BNMN levels are somewhat more inconsistent, and many studies failing to find an association except, maybe, in heavy smokers and in those with relevant occupational exposures [48,49,50,51]. In this study, no significant effect of gender, age, or smoking habits on BNMN levels or its net balance was detected, irrespective of the time point or dose group. The study was probably underpowered to detect such effects. It is also possible that the effect of these variables may have been masked by the impact of internal IR exposure after ^131^I administration.

We did observe, however, in the 70 mCi group only, differences on BNMN levels between the two TC histotypes, as FTC patients presented significantly higher basal BNMN frequency than PTC patients but significantly lower therapy-induced BNMN levels at one month after ^131^I administration. This is suggestive of higher background genomic instability in FTC but higher sensitivity to the DNA damaging effects of IR in PTC. Considering the small sample size and the non-reproducibility of the findings between the two dose groups, extreme caution must be taken in the interpretation of these results. Nevertheless, the available evidence supports both findings: PTC usually presents as a microsatellite stable tumor, with no appreciable levels of either loss of heterozygosity (LOH) or aneuploidy (stable chromosome profile) [52,53,54]. On the contrary, a considerable degree of chromosomal instability appears to be a hallmark feature of FTC, which presents a consistently higher frequency of chromosomal abnormalities, LOH, allelic loss, and a higher mutational burden compared to PTC [52,53,55,56,57]. Microsatellite instability (MSI), despite uncommon in TC, also appears to be more frequent in FTC than in PTC [53,54,55]. The available evidence thus largely supports our observation of higher background genomic instability in FTC. Moreover, considering that activating *RAS* mutations are commonly observed in FTC but not in PTC [53,58,59], the association between increased *RAS* expression and decreased frequency of IR-induced MN reported by Miller et al. [60] is coherent with our own observation of lower ^131^I-induced BNMN frequency in FTC, supporting the idea that this histotype is less sensitive to the DNA damaging effects of IR than PTC. Such hypothesis (i.e., higher sensitivity to IR in PTC) is further reinforced by a recent observation, through meta-analysis, of increased efficacy of RAI therapy in PTC patients, compared to FTC [61] but more studies are needed for a solid conclusion to be drawn.

Moreover, in the present study, we further evaluated the potential impact of selected HR, NHEJ, and MMR pathway SNPs on BNMN levels, before and after the administration of ^131^I. To our knowledge, this is the first study doing so. Significant genotype effects on MN frequency and/or its net balance were observed for HR (*NBN*) and MMR (*MLH1*, *MSH3*, *MSH4*) repair pathway SNPs across different time points. This was expected because (1) IR exposure results in increased DNA damage, most notably, single- and double-strand breaks, oxidative lesions (e.g., 8-oxoG), DNA-protein crosslinks (DPCs) and clustered DNA lesions [62,63,64,65,66,67]; (2) the HR pathway, acting in the S/G2 stages of the cell cycle, is the major DNA repair pathway involved in the error-free correction of DSBs [11,33,35,68]; (3) MMR proteins, besides their canonical actions on the post-replication repair of mispaired nucleotides and insertion–deletion loops, have also been demonstrated to play an important role on the damage response to IR-induced DSBs, either through cooperation with HR or through signaling for cell-cycle arrest and apoptosis [64,69,70,71]; (4) DSBs, if left unrepaired, e.g., due to the presence of SNPs that reduce the DNA repair capacity, may give rise to chromosome breakage and MN formation upon replication [28,33,35,72]. The potential influence of functional DSB repair SNPs on ^131^I-induced BNMN frequency is, therefore, fully justified. A literature review on the functional impact of these SNPs and their putative association with response to radio and/or chemotherapy was performed and is presented below (Table 6).

MLH1, together with PMS2, forms the MutLα heterodimer, a complex critical for the maintenance of genomic integrity [103,104]. The common rs1799977 (c.665A>G, Ile219Val) missense SNP is located in a region that codes for a highly conserved N-terminal ATPase domain, vital for MLH1 function. However, since both alleles code for nonpolar pH-neutral amino acids, the substitution is considered conservative and not expected to result in drastic changes in protein properties and function [73]. Several functional studies support this hypothesis [73,74,105,106,107] but the existence of a more subtle effect should not be excluded [73,106,108,109] as an association between the G variant allele and reduced MLH1 expression has been demonstrated repeatedly in cancer patients [74,75,76,77]. Moreover, two recent meta-analyses have associated this variant with increased risk of colorectal cancer [110,111]. Considering the important role that MLH1 plays in the maintenance of genome integrity and cancer avoidance, both observations are compatible with our own observation of increased baseline BNMN levels in TC patients carrying the G allele. A different picture emerges, however, upon IR exposure: as previously stated, MMR proteins such as MLH1 play a dual role in the DNA damage response to IR, triggering cell-cycle arrest and allowing for either DSB repair or apoptosis [11,64]. MMR proficiency is thus expected to result in higher repair efficiency of IR-induced damage (hence, lower cytogenetic levels) and, simultaneously, higher cytotoxicity upon IR exposure (hence, increased sensitivity to radiotherapy). Indeed, alongside with increased cancer susceptibility, the *MLH1* rs1799977 variant GG genotype has been associated with increased radiosensitivity in cancer patients, translating into increased efficacy [78] or toxicity [79] of radiotherapy (alone or combined with chemotherapy). This is suggestive of increased MMR proficiency in such patients and supports our own observation of significantly lower BNMN levels, one month after ^131^I therapy, in TC patients carrying the G allele. How the same allele may be associated with decreased function under basal conditions and increased function after IR exposure remains to be explained: MLH1 has been demonstrated to be upregulated upon IR exposure [112,113], it is possible that such upregulation might be more pronounced in G allele carriers, but this is highly speculative. Nevertheless, the high level of significance in our observations (especially when considering the change in MN frequency from baseline) and their cross-validation in independent groups strengthen our conclusions and warrant further studies to clarify this issue.

Two other MMR polymorphisms presented significant findings in our study, *MSH3* rs26279 and *MSH4* rs5745325. Like MLH1, MSH3 also appears to be involved in the repair and damage response to IR-associated lesions such as DSBs and inter-strand crosslinks [84,114]. *MSH3* rs26279 (c.3133A>G; Thr1045Ala) is a common SNP that results in an amino acid change in the ATPase domain of MLH3. This domain is critical for MSH3 activity, suggesting a functional impact for this variant [80]. Such hypothesis remains to be verified as, to the best of our knowledge, functional studies are lacking. An association with altered MSH3 expression levels has been suggested [81] but not confirmed [82]. The *MSH3* rs26279 G allele or GG genotype has been consistently associated with cancer risk in all 3 meta-analysis that we are aware of, particularly for colon and breast cancer [115,116,117], suggesting decreased DNA repair capacity in G allele carriers. Further, *MSH3* rs26279 GG homozygosity has also been associated with decreased incidence of radiation dermatitis in breast cancer patients receiving radiotherapy [83], decreased overall survival in head and neck squamous cell carcinoma patients submitted to radiochemotherapy [81], and decreased response to platinum-based chemotherapy in advanced non-small cell lung cancer patients [84], suggesting decreased sensitivity to DNA damaging agents such as IR or platinum in GG homozygous individuals. Such phenotype is commonly associated with MMR deficiency [64,69,70,118,119]. If we consider, once again, the dual role that MMR proteins such as MSH3 play in damage repair and apoptosis, these results are compatible with decreased G allele function, resulting in decreased DNA repair and apoptosis, increased damage tolerance, resistance to radio/chemotherapy, and reduced efficacy and cytotoxicity of such therapeutic agents. Our own observation of increased MN levels in TC patients carrying the G allele, 6 months after receiving 100 mCi ^131^I, fits comfortably into this picture.

Likewise, in our study, MN frequency was also significantly increased (absolute and change from baseline values) in TC patients carrying the A allele of *MSH4* rs5745325, one month after ^131^I administration. *MSH4* rs5745325 (c.289G>A; Ala97Thr) has only seldom been evaluated: on single SNP analysis, two prior studies by our team failed to detect an association with either thyroid [21] or breast cancer risk [120]. The same was observed in the only two other association studies that we found focusing on this SNP [121,122]. Interestingly, in three out of these four studies, significant associations were detected when interactions with other SNPs—*MSH6* rs1042821 [21], *MLH3* rs175080 [120], and *CHRNA5* rs16969968 [121]—were considered. Besides the important role that MSH4 plays in recombinational repair during meiosis [123], it is also suggested to participate, through interaction with a vast array of binding partners, in DSB-triggered damage response and repair [85,123,124]. It is possible that *MSH4* rs5745325 interferes with the binding properties of MSH4, with impact on its putative contribution to the DNA damage response and repair. The interaction of MSH4 with eIF3f (a subunit of the eIF3 complex implicated in apoptosis regulation and tumor development), for example, occurs at the region comprising the first 150 amino acids of the N-terminal domain of MSH4 (where rs5745325 is located) and has been demonstrated to foster hMSH4 stabilization and to modulate sensitivity to IR-induced DNA damage [85]. This is in line with our own findings.

Finally, we also observed a significant association between *NBN* rs1805794 and BNMN frequency, one month after the administration of 100 mCi ^131^I. Nibrin plays a pivotal role in the initial steps of the cellular response to DNA damage, directly initiating DSB repair through the RAD51-dependent HR pathway and further contributing to cell cycle checkpoint activation through an ATM-dependent pathway [68,125,126,127]. Inactivating germline mutations in the *NBN* gene (which encodes for the Nibrin protein) markedly impair DSB repair and cause the Nijmegen breakage syndrome, characterized by chromosomal instability, increased cancer susceptibility, and increased sensitivity to DSB-causing agents such as IR or cisplatin. These features highlight the importance of Nibrin for genome stability (hence, cancer prevention) [86,93,125,127]. NBN overexpression also appears to be associated with poor prognosis in several types of cancer [68], which is consistent with a putative increase in DNA repair efficiency, hence, resistance to cytotoxic therapy. Among the numerous *NBN* polymorphisms, rs1805794 (c.553G>C; Glu185Gln) is the most frequently investigated. This missense variant results in an amino acid change in the BRCT (BRCA1 C Terminus) domain (amino acids 108-196), a domain involved in the interaction of Nibrin with BRCA1. The resulting complex (the BRCA1-associated genome surveillance complex, BASC) is responsible for the recognition and repair of aberrant DNA [86,87,88,89]. *NBN* rs1805794 has been suggested to interfere with the interaction properties of Nibrin and thus with DNA repair capacity, sensitivity to DNA damaging agents (such as IR) and cancer susceptibility. Accordingly, *NBN* rs1805794 has been repeatedly associated with cancer risk, as demonstrated by numerous meta-analysis [68,88,89,125,128,129,130,131,132] but conflicting reports exist [126,127,133,134]. Interestingly, the association may vary according to ethnicity [88,130] and tumor site [125], as one of these meta-analysis has demonstrated, for example, increased risk of leukemia, nasopharyngeal, and urinary system cancers but decreased risk of lung, gastric, and digestive system cancers [125]. Furthermore, final conclusive evidence on the significance of *NBN* rs1805794 is still lacking, as the functional studies performed thus far have yielded negative or conflicting results: while lymphocytes from healthy individuals homozygous for the G allele have been reported to present higher DNA damage levels (as assessed by the Comet assay) than lymphocytes from C allele carriers [90], opposite results have been reported in ex vivo X-ray irradiated cells from healthy subjects [88]. Further ex vivo irradiation studies have failed to observe a significant influence of *NBN* rs1805794 on DNA repair capacity and radiosensitivity [91,92]. Furthermore, since a putative functional impact of this SNP on DNA repair capacity could possibly influence patient sensitivity to radio and/or chemotherapy, association studies correlating *NBN* rs1805794 genotype with therapy response, toxicity, or prognosis have also been performed. Again, most studies failed to find an association in radiotherapy [79,93,94,95,96] or chemotherapy [97,98,99] treated patients, while other studies presented opposite findings, associating the *NBN* rs1805794 C allele with either improved [86,100] or worse [68,101] prognosis upon platinum-based chemotherapy. Interestingly, increased frequency of binucleated lymphocytes with nucleoplasmic bridges was observed in peripheral lymphocytes from children with high environmental exposure to IR that were heterozygous for *NBN* rs1805794, while the reverse patter was observed in children homozygous for the Gln allele [102]. This may be suggestive of molecular heterosis, a hypothesis that, considering the high interethnic variability of the *NBN* rs1805794 distribution, could help in explaining such divergent results. Overall, despite extensively investigated, the functional significance of *NBN* rs1805794, as well as its putative role in sensitivity to DNA damaging agents (such as IR) and cancer susceptibility remains elusive, warranting further studies to clarify this issue.

## 5. Conclusions

In conclusion, our results confirm that BNMN levels in peripheral lymphocytes from DTC patients increase significantly immediately 1 month after ^131^I therapy and further suggest that these remain stable and persistently higher than baseline for at least 2 years. Furthermore, a marked reduction in CBPI is observed at 24 months after ^131^I administration. Moreover, HR and MMR SNPs (*MLH1* rs1799977, *MSH3* rs26279, *MSH4* rs5745325, and *NBN* rs1805794) were, for the first time, associated with IR-induced MN, a cytogenetic marker of DNA damage, in TC patients submitted to ^131^I therapy. Among such findings, a highly significant and independently replicated association was observed for *MLH1* rs1799977, strongly suggesting a role for this particular SNP on the personalization of RAI therapy in TC cancer patients. Baseline and post-therapy MN levels also diverged according to tumor histotype. These results should be regarded as merely suggestive and proof of concept, as the sample was small and the number of tests was high, increasing the likelihood of false-positive results. Nevertheless, our findings suggest that TC therapy with ^131^I may pose a long-term challenge to cells other than thyrocytes and that the patient genetic profile may influence the individual sensitivity to this therapy. Such hypotheses are of relevance to the efficacy and safety of ^131^I therapy, a widespread practice in TC patients. As such, extending the benefit already achieved with the latest guidelines on TC treatment in terms of risk/benefit ratio through improved clinical assessment of the potential long-term risks of ^131^I therapy is desirable. Likewise, despite the micronucleus test is considered the gold standard methodology in genetic toxicology testing and often used as a “stand-alone” test in numerous and relevant papers in this area, other tests should also be employed to validate these results. Furthermore, potential radiogenomic markers such as those suggested here should be evaluated in larger samples, preferentially through multi-center independent studies adequately powered to provide more robust evidence and, eventually, to allow for gene-gene and gene-environment interactions to be assessed. Identifying the most clinically relevant variables, genetic or non-genetic, and accurately estimating their impact on ^131^I therapy response rate and adverse event risk for each individual TC patient is the ultimate goal, under a personalized medicine approach.

## Figures and Tables

**Table 1:** Selected SNPs and detailed information on the corresponding base and amino acid changes, minor allele frequency, and Applied Biosystems (AB) assay used for genotyping.

Gene	Location	DB SNP Cluster ID (RS NO.)	Base Change	Amino Acid Change	MAF (%) ^a^	AB Assay ID
*MLH1*	3p22.2	rs1799977	A → G	Ile219Val	23.3	C___1219076_20
*MSH3*	5q14.1	rs26279	A → G	Thr1045Ala	27.1	C____800002_1_
*MSH4*	1p31.1	rs5745325	G → A	Ala97Thr	26.0	C___3286081_10
*PMS1*	2q32.2	rs5742933	G → C	-- ^b^	23.4	C__29329633_10
*MSH6*	2p16.3	rs1042821	C → T	Gly39Glu	18.2	C___8760558_10
*RAD51*	15q15.1	rs1801321	G → T	-- ^b^	33.2	C___7482700_10
*NBN*	8q21.3	rs1805794	G → C	Glu185Gln	34.7	C__26470398_30
*XRCC3*	14q32.33	rs861539	C → T	Thr241Met	29.0	-- ^d^
*XRCC5*	2q35	rs2440	C → T	-- ^c^	36.3	C___3231046_10

^a^ MAF, minor allele frequency, according to the Genome Aggregation Database (gnomAD), v2.1.1, available at https://gnomad.broadinstitute.org/. ^b^ SNP located on 5′ UTR. ^c^ SNP located on 3′ UTR. ^d^ not applicable (genotyping performed by PCR-RFLP). SNPs, single nucleotide polymorphisms.

**Table 2:** General characteristics for differentiated thyroid carcinoma (DTC) patients treated with 70 mCi (*n* = 15) and 100 mCi (*n* = 11) ^131^I.

Characteristics	Study Population *n* (%)	70 mCi *n* (%)	100 mCi *n* (%)	*p* Value ^c^
**Gender**				
Male	3 (11.5)	1 (6.7)	2 (18.2)	0.556
Female	23 (88.5)	14 (93.3)	9 (81.8)	
**Age ^a^**	52.54 ± 11.62 ^b^	52.07 ± 10.26 ^b^	53.18 ± 13.76 ^b^	0.815
≤54	14 (53.8)	8 (53.3)	6 (54.5)	1.000
>54	12 (46.2)	7 (46.7)	5 (45.5)	
**Smoking habits**				
Non-smokers	22 (84.6)	13 (86.7)	9 (81.8)	1.000
Smokers	4 (15.4)	2 (13.3)	2 (18.2)	
**Histology**				
Papillary	18 (69.2)	10 (66.7)	8 (72.7)	1.000
Follicular	8 (30.8)	5 (33.3)	3 (27.3)	

^a^ For age categorization purposes, the median age of all patients included in the study (54 years) was defined as the cut-off point. ^b^ mean ± S.D. ^c^ *p* value for 70 mCi *versus* 100 mCi groups determined by two-sided Fisher’s exact test (gender, smoking habits, and age categories) or Student *t* test (age mean ± S.D.).

**Table 3:** Allele and genotype frequencies in DTC patients submitted to ^131^I therapy.

Genotype	70 mCi (*n* = 15)		100 mCi (*n* = 11)		TOTAL (*n* = 26)	
	MAF	Genotype Frequency *n* (%)	MAF	Genotype Frequency *n* (%)	MAF	Genotype Frequency *n* (%)
*MLH1* rs1799977						
Ile/Ile	G: 0.30	7 (46.7)	G: 0.45	3 (27.3)	G: 0.37	10 (38.5)
Ile/Val		7 (46.7)		6 (54.5)		13 (50.0)
Val/Val		1 (6.7)		2 (18.2)		3 (11.5)
Ile/Val+Val/Val		8 (53.3)		8 (72.7)		16 (61.5)
*MSH3* rs26279						
Thr/Thr	G: 0.23	10 (66.7)	G: 0.14	8 (72.7)	G: 0.19	18 (69.2)
Thr/Ala		3 (20.0)		3 (27.3)		6 (23.1)
Ala/Ala		2 (13.3)		0 (0.0)		2 (7.7)
Thr/Ala+Ala/Ala		5 (33.3)		3 (27.3)		8 (30.8)
*MSH4* rs5745325						
Ala/Ala	A: 0.13	11 (73.3)	A: 0.32	4 (36.4)	A: 0.21	15 (57.7)
Ala/Thr		4 (26.7)		7 (63.6)		11 (42.3)
Thr/Thr		0 (0.0)		0 (0.0)		0 (0.0)
Ala/Thr+Thr/Thr		4 (26.7)		7 (63.6)		11 (42.3)
*PMS1* rs5742933						
G/G	C: 0.18	10 (71.4)	C: 0.14	9 (81.8)	C: 0.16	19 (76.0)
G/C		3 (21.4)		1 (9.1)		4 (16.0)
C/C		1 (7.1)		1 (9.1)		2 (8.0)
G/C+C/C		4 (28.6)		2 (18.2)		6 (24.0)
*MSH6* rs1042821						
Gly/Gly	T: 0.17	10 (66.7)	T: 0.09	9 (81.8)	T: 0.13	19 (73.1)
Gly/Glu		5 (33.3)		2 (18.2)		7 (26.9)
Glu/Glu		0 (0.0)		0 (0.0)		0 (0.0)
Gly/Glu+Glu/Glu		5 (33.3)		2 (18.2)		7 (26.9)
*RAD51* rs1801321						
T/T	G: 0.50	4 (26.7)	G: 0.45	4 (36.4)	G: 0.48	8 (30.8)
T/G		7 (46.7)		4 (36.4)		11 (42.3)
G/G		4 (26.7)		3 (27.3)		7 (26.9)
T/G+G/G		11 (73.3)		7 (63.6)		18 (69.2)
*NBN* rs1805794						
Glu/Glu	C: 0.30	7 (46.7)	C: 0.14	8 (72.7)	C: 0.23	15 (57.7)
Glu/Gln		7 (46.7)		3 (27.3)		10 (38.5)
Gln/Gln		1 (6.7)		0 (0.0)		1 (3.8)
Glu/Gln+Gln/Gln		8 (53.3)		3 (27.3)		11 (42.3)
*XRCC3* rs861539						
Thr/Thr	C: 0.47	5 (33.3)	T: 0.36	5 (45.5)	T: 0.46	10 (38.5)
Thr/Met		4 (26.7)		4 (36.4)		8 (30.8)
Met/Met		6 (40.0)		2 (18.2)		8 (30.8)
Thr/Met+Met/Met		10 (66.7)		6 (54.5)		16 (61.5)
*XRCC5* rs2440						
T/T	C: 0.47	5 (33.3)	C: 0.50	2 (22.2)	C: 0.48	7 (29.2)
T/C		6 (40.0)		5 (55.6)		11 (45.8)
C/C		4 (26.7)		2 (22.2)		6 (25.0)
T/C+C/C		10 (66.7)		7 (77.8)		17 (70.8)

MAF, minor allele frequency. All comparisons of genotype distributions were performed by the two-sided Fisher’s exact test (whenever 2 × 2 contingency tables are possible) or the χ^2^ test (remaining cases). No significant differences among the 70 and 100 mCi dose groups were observed.

**Table 4:** Frequency of micronucleated cells (‰BNMN, mean ± SD) in each ^131^I dose group at t_0_, t_1_, t_3_/t_6,_ and t_24_, according to genotype (only SNPs presenting significant findings are shown).

Genotype	70 mCi Group (*n* = 15), ‰BNMN (Mean ± SD)				100 mCi Group (*n* = 11), ‰BNMN (Mean ± SD)			70 + 100 mCi Groups (*n* = 26), ‰BNMN (Mean ± SD)	
	t_0_	t_1_	t_6_	t_24_	t_0_	t_1_	t_3_	t_0_	t_1_
*MLH1* rs1799977									
Ile/Ile	4.14 ± 3.29	**12.14 ± 3.58**	10.86 ± 7.11	9.20 ± 1.30	**5.33 ± 1.16**	**24.00 ± 3.46**	21.50 ± 7.78	**4.50 ± 2.80**	**15.70 ± 6.63**
Ile/Val + Val/Val	6.25 ± 3.85	5.88 ± 3.36 *	7.25 ± 4.46	10.00 ± 3.74	11.25 ± 4.62 *	14.75 ± 2.77 *	21.38 ± 5.71	8.75 ± 4.85 *	10.31 ± 5.46 *
*MSH3* rs26279									
Thr/Thr	5.50 ± 3.63	8.90 ± 3.81	9.90 ± 7.09	10.13 ± 1.64	8.00 ± 2.73	16.88 ± 5.79	**19.00 ± 4.93**	6.61 ± 3.42	12.44 ± 6.18
Thr/Ala + Ala/Ala	4.80 ± 4.03	8.60 ± 6.54	7.00 ± 1.58	8.33 ± 5.13	14.00 ± 7.00	18.33 ± 3.51	27.00 ± 2.00 *	8.25 ± 6.78	12.25 ± 7.31
*MSH4* rs5745325									
Ala/Ala	5.18 ± 3.79	8.91 ± 5.07	9.09 ± 6.64	9.63 ± 3.34	13.25 ± 5.68	13.75 ± 3.50	25.50 ± 4.73	7.33 ± 5.55	**10.20 ± 5.09**
Ala/Thr + Thr/Thr	5.50 ± 3.70	8.50 ± 3.87	8.50 ± 4.04	9.67 ± 0.58	7.57 ± 2.88	19.29 ± 4.99	18.67 ± 4.68	6.82 ± 3.19	15.36 ± 7.00 *
*NBN* rs1805794									
Glu/Glu	5.43 ± 4.61	10.00 ± 4.51	8.14 ± 4.56	9.86 ± 2.12	9.00 ± 4.84	**19.13 ± 4.64**	19.57 ± 4.89	7.33 ± 4.92	**14.87 ± 6.46**
Glu/Gln + Gln/Gln	5.13 ± 2.85	7.75 ± 4.80	9.63 ± 7.15	9.25 ± 4.11	11.33 ± 5.13	12.33 ± 2.52 *	25.67 ± 5.77	6.82 ± 4.40	9.00 ± 4.69 *

* *p* < 0.05; *p*-value for variant allele carriers *versus* common allele homozygotes determined by the Student *t* test (whenever a normal distribution could not be excluded through the Shapiro-Wilk test) or the Mann-Whitney U test (remaining cases). Significant findings highlighted in bold.

**Table 5:** Variation in the frequency of micronucleated cells from baseline (‰BNMN, mean ± SD) in each ^131^I dose group at t_1_, t_3_/t_6,_ and t_24_, according to genotype (only SNPs presenting significant findings are shown).

Genotype	70 mCi Group (*n* = 15), ‰BNMN (mean ± SD)			100 mCi Group (*n* = 11), ‰BNMN (mean ± SD)		70 + 100 mCi Groups (*n* = 26), ‰BNMN (mean ± SD)
	Δt_1_	Δt_6_	Δt_24_	Δt_1_	Δt_3_	Δt_1_
*MLH1* rs1799977						
Ile/Ile	**8.00 ± 4.97**	6.71 ± 6.85	5.00 ± 3.39	**18.67 ± 3.06**	16.50 ± 6.36	**11.20 ± 6.71**
Ile/Val + Val/Val	−0.38 ± 3.70 *	1.00 ± 4.90	3.50 ± 4.37	3.50 ± 4.57 *	10.13 ± 5.28	1.56 ± 4.49 *
*MSH4* rs5745325						
Ala/Ala	3.73 ± 6.83	3.91 ± 7.05	4.13 ± 3.91	**0.50 ± 3.11**	12.25 ± 5.32	**2.87 ± 6.13**
Ala/Thr + Thr/Thr	3.00 ± 3.56	3.00 ± 4.90	4.33 ± 4.51	11.71 ± 7.27 *	10.83 ± 6.49	8.55 ± 7.41 *

* *p* < 0.05; *p*-value for variant allele carriers *versus* common allele homozygotes determined by the Student *t* test (whenever a normal distribution could not be excluded through the Shapiro-Wilk test) or the Mann-Whitney U test (remaining cases). Significant findings highlighted in bold.

**Table 6:** Literature review on the functional impact of the studied SNPs and their putative association with radio and/or chemosensitivity (only SNPs presenting significant findings in the present study are shown).

Gene	DB SNP Cluster ID (RS NO.)	Functional Impact	Clinical Association Studies (Radio and/or Chemosensitivity)
*MLH1*	rs1799977	Missense SNP located in a highly conserved N-terminal ATPase domain, vital for MLH1 function [73]; G allele associated with reduced expression [74,75,76,77].	GG genotype associated with increased radiosensitivity in cancer patients, translating into increased efficacy [78] or toxicity [79] of radiotherapy (alone or combined with chemotherapy).
*MSH3*	rs26279	Missense SNP located in the ATPase domain, critical for protein activity [80]; altered expression has been suggested [81] but not confirmed [82].	GG genotype associated with decreased incidence of radiation dermatitis in breast cancer patients receiving radiotherapy [83], decreased overall survival in head and neck squamous cell carcinoma patients submitted to radiochemotherapy [81] and decreased response to platinum-based chemotherapy in advanced non-small cell lung cancer patients [84].
*MSH4*	rs5745325	Missense SNP located in the N-terminal domain, involved in the interaction with eIF3f [85].	None to be reported.
*NBN*	rs1805794	Missense SNP located in the BRCT domain, a region involved in the interaction with BRCA1 [86,87,88,89]; conflicting results from functional studies [88,90,91,92].	No association detected in most studies focusing on response to radiotherapy [79,93,94,95,96] or chemotherapy [97,98,99]; conflicting results also reported as the C allele has been associated with either improved [86,100] or worse [68,101] prognosis upon platinum-based chemotherapy; increased frequency of binucleated lymphocytes with nucleoplasmic bridges in Glu/Gln children with high IR exposure, opposite to Gln/Gln children [102].

## Supplementary Materials

The following are available online at https://www.mdpi.com/2073-4425/11/9/1083/s1, Table S1: Allele and genotype frequencies in thyroid cancer patients submitted to ^131^I therapy (*n* = 26) and in the original (reference) DTC population (*n* = 106), Table S2: BNMN frequency (‰, mean ± S.D.) in DTC patients before and after (1, 3/6, and 24 months) therapy with different doses of ^131^I (70 and 100 mCi), Table S3: Frequency of micronucleated cells (‰BNMN, mean ± SD) in the 70 mCi dose group at t_0_, t_1_, t_6_ and t_24_, and corresponding variation, according to genotype, Table S4: Frequency of micronucleated cells (‰BNMN, mean ± SD) in the 100 mCi dose group at t_0_, t_1_ and t_3_, and corresponding variation, according to genotype, Table S5: Frequency of micronucleated cells (‰BNMN, mean ± SD) in the combined dose groups at t_0_ and t_1_, and corresponding variation, according to genotype, Table S6: Cytokinesis-Block Proliferation Index (CBPI, mean ± SD) in the 70 mCi dose group at t_0_, t_1_, t_6_ and t_24_, and corresponding variation, according to genotype.
