# Supplementary material for: Micronuclei Formation upon Radioiodine Therapy for Well-Differentiated Thyroid Cancer: The Influence of DNA Repair Genes Variants
Source: Genes (Basel). 2020 Sep 17;11(9):1083. doi: 10.3390/genes11091083 (PMC7565468; doi:10.3390/genes11091083)
Supplement: Supplementary file 1 [file genes-11-01083-s001.pdf]

Table S1 – Allele and genotype frequencies in thyroid cancer patients submitted to ^{131}I therapy (n=26) and in the reference DTC population (n=106).

Genotype	Reference DTC population (n=106)		Study sample					
			70 mCi (n=15)		100 mCi (n=11)		TOTAL (n=26)	
	MAF	Genotype frequency n (%)	MAF	Genotype frequency n (%)	MAF	Genotype frequency n (%)	MAF	Genotype frequency n (%)
MLH1 **rs1799977**								
Ile/Ile	G: 0.30	48 (45.7)	G: 0.30	7 (46.7)	G: 0.45	3 (27.3)	G: 0.37	10 (38.5)
Ile/Val		50 (47.6)		7 (46.7)		6 (54.5)		13 (50.0)
Val/Val		7 (6.7)		1 (6.7)		2 (18.2)		3 (11.5)
Ile/Val+Val/Val		57 (54.3)		8 (53.3)		8 (72.7)		16 (61.5)
MSH3 **rs26279** [a]								
Thr/Thr	G: 0.33	48 (45.7)	G: 0.23	10 (66.7)	G: 0.14	8 (72.7)	G: 0.19	18 (69.2)
Thr/Ala		45 (42.9)		3 (20.0)		3 (27.3)		6 (23.1)
Ala/Ala		12 (11.4)		2 (13.3)		0 (0.0)		2 (7.7)
Thr/Ala+Ala/Ala		57 (54.3)		5 (33.3)		3 (27.3)		8 (30.8)
MSH3 **rs184967**								
Arg/Arg	A: 0.15	80 (75.5)	A: 0.10	13 (86.7)	A: 0.14	8 (72.7)	A: 0.12	21 (80.8)
Arg/Gln		21 (19.8)		1 (6.7)		3 (27.3)		4 (15.4)
Gln/Gln		5 (4.7)		1 (6.7)		0 (0.0)		1 (3.8)
Arg/Gln+Gln/Gln		26 (24.5)		2 (13.3)		3 (27.3)		5 (19.2)
MSH4 **rs5745549**								

Ser/Ser	A: 0.07	92 (86.8)	A: 0.03	14 (93.3)	A: 0.14	8 (72.7)	A: 0.08	22 (84.6)
Ser/Asn		14 (13.2)		1 (6.7)		3 (27.3)		4 (15.4)
Asn/Asn		0 (0.0)		0 (0.0)		0 (0.0)		0 (0.0)
Ser/Asn+Asn/Asn		14 (13.2)		1 (6.7)		3 (27.3)		4 (15.4)
***MSH4* rs5745325**								
Ala/Ala	A: 0.27	57 (53.8)	A: 0.13	11 (73.3)	A: 0.32	4 (36.4)	A: 0.21	15 (57.7)
Ala/Thr		40 (37.7)		4 (26.7)		7 (63.6)		11 (42.3)
Thr/Thr		9 (8.5)		0 (0.0)		0 (0.0)		0 (0.0)
Ala/Thr+Thr/Thr		49 (46.2)		4 (26.7)		7 (63.6)		11 (42.3)
***PMS1* rs5742933**								
G/G	C: 0.17	73 (70.2)	C: 0.18	10 (71.4)	C: 0.14	9 (81.8)	C: 0.16	19 (76.0)
G/C		27 (26.0)		3 (21.4)		1 (9.1)		4 (16.0)
C/C		4 (3.8)		1 (7.1)		1 (9.1)		2 (8.0)
G/C+C/C		31 (29.8)		4 (28.6)		2 (18.2)		6 (24.0)
***MLH3* rs175080** [b]								
Leu/Leu	G: 0.49	25 (23.6)	G: 0.30	7 (46.7)	A: 0.32	1 (9.1)	G: 0.46	8 (30.8)
Leu/Pro		59 (55.7)		7 (46.7)		5 (45.5)		12 (46.2)
Pro/Pro		22 (20.8)		1 (6.7)		5 (45.5)		6 (23.1)
Leu/Pro+ Pro/Pro		81 (76.4)		8 (53.3)		10 (90.9)		18 (69.2)
***MSH6* rs1042821**								
Gly/Gly	T: 0.22	68 (64.2)	T: 0.17	10 (66.7)	T: 0.09	9 (81.8)	T: 0.13	19 (73.1)
Gly/Glu		30 (28.3)		5 (33.3)		2 (18.2)		7 (26.9)
Glu/Glu		8 (7.5)		0 (0.0)		0 (0.0)		0 (0.0)
Gly/Glu+Glu/Glu		38 (35.8)		5 (33.3)		2 (18.2)		7 (26.9)

***RAD51* rs1801321**								
T/T	G: 0.50	28 (26.4)	G: 0.50	4 (26.7)	G: 0.45	4 (36.4)	G: 0.48	8 (30.8)
T/G		50 (47.2)		7 (46.7)		4 (36.4)		11 (42.3)
G/G		28 (26.4)		4 (26.7)		3 (27.3)		7 (26.9)
T/G+G/G		78 (73.6)		11 (73.3)		7 (63.6)		18 (69.2)
***NBN* rs1805794**								
Glu/Glu	C: 0.28	55 (51.9)	C : 0.30	7 (46.7)	C : 0.14	8 (72.7)	C : 0.23	15 (57.7)
Glu/Gln		43 (40.6)		7 (46.7)		3 (27.3)		10 (38.5)
Gln/Gln		8 (7.5)		1 (6.7)		0 (0.0)		1 (3.8)
Glu/Gln+Gln/Gln		51 (48.1)		8 (53.3)		3 (27.3)		11 (42.3)
***XRCC2* rs3218536**								
Arg/Arg	A: 0.07	92 (86.8)	A: 0.10	12 (80.0)	A: 0.00	11 (100.0)	A: 0.06	23 (88.5)
Arg/His		14 (13.2)		3 (20.0)		0 (0.0)		3 (11.5)
His/His		0 (0.0)		0 (0.0)		0 (0.0)		0 (0.0)
Arg/His+His/His		14 (13.2)		3 (20.0)		0 (0.0)		3 (11.5)
***XRCC3* rs861539**								
Thr/Thr	T: 0.45	36 (34.0)	C: 0.47	5 (33.3)	T: 0.36	5 (45.5)	T: 0.46	10 (38.5)
Thr/Met		44 (41.5)		4 (26.7)		4 (36.4)		8 (30.8)
Met/Met		26 (24.5)		6 (40.0)		2 (18.2)		8 (30.8)
Thr/Met+Met/Met		70 (66.0)		10 (66.7)		6 (54.5)		16 (61.5)
***XRCC4* rs1805377**								
G/G	A: 0.08	90 (84.9)	A: 0.10	12 (80.0)	A: 0.05	10 (90.9)	A: 0.08	22 (84.6)
G/A		15 (14.2)		3 (20.0)		1 (9.1)		4 (15.4)
A/A		1 (0.9)		0 (0.0)		0 (0.0)		0 (0.0)

G/A+A/A		16 (15.1)		3 (20.0)		1 (9.1)		4 (15.4)
***LIG4* rs1805388**								
Thr/Thr	T: 0.14	81 (76.4)	T: 0.10	12 (80.0)	T: 0.09	9 (81.8)	T: 0.10	21 (80.8)
Thr/Ile		21 (19.8)		3 (20.0)		2 (18.2)		5 (19.2)
Ile/Ile		4 (3.8)		0 (0.0)		0 (0.0)		0 (0.0)
Thr/Ile+Ile/Ile		25 (23.6)		3 (20.0)		2 (18.2)		5 (19.2)
***XRCC4* rs28360135**								
Ile/Ile	C: 0.06	94 (88.7)	C: 0.07	13 (86.7)	C: 0.00	11 (100.0)	C: 0.04	24 (92.3)
Ile/Thr		12 (11.3)		2 (13.3)		0 (0.0)		2 (7.7)
Thr/Thr		0 (0.0)		0 (0.0)		0 (0.0)		0 (0.0)
Ile/Thr+Thr/Thr		12 (11.3)		2 (13.3)		0 (0.0)		2 (7.7)
***XRCC5* rs1051685**								
A/A	G: 0.09	89 (84.0)	G: 0.03	14 (93.3)	G: 0.09	9 (81.8)	G: 0.06	23 (88.5)
A/G		15 (14.2)		1 (6.7)		2 (18.2)		3 (11.5)
G/G		2 (1.9)		0 (0.0)		0 (0.0)		0 (0.0)
A/G+G/G		17 (16.0)		1 (6.7)		2 (18.2)		3 (11.5)
***XRCC5* rs1051677**								
T/T	C: 0.13	79 (74.5)	C: 0.17	11 (73.3)	C: 0.18	7 (63.6)	C: 0.17	18 (69.2)
T/C		26 (24.5)		3 (20.0)		4 (36.4)		7 (26.9)
C/C		1 (0.9)		1 (6.7)		0 (0.0)		1 (3.8)
T/C+C/C		27 (25.5)		4 (26.7)		4 (36.4)		8 (30.8)
***XRCC5* rs6941**								
C/C	A: 0.13	78 (75.0)	A: 0.17	11 (73.3)	A: 0.17	6 (66.7)	A: 0.17	17 (70.8)
C/A		25 (24.0)		3 (20.0)		3 (33.3)		6 (25.0)

A/A		1 (1.0)		1 (6.7)		0 (0.0)		1 (4.2)
C/A+A/A		26 (25.0)		4 (26.7)		3 (33.3)		7 (29.2)
XRCC5 **rs2440**								
T/T	T: 0.45	20 (19.2)	C: 0.47	5 (33.3)	C: 0.50	2 (22.2)	C: 0.48	7 (29.2)
T/C		54 (51.9)		6 (40.0)		5 (55.6)		11 (45.8)
C/C		30 (28.8)		4 (26.7)		2 (22.2)		6 (25.0)
T/C+C/C		84 (80.8)		10 (66.7)		7 (77.8)		17 (70.8)

All comparisons of genotype distributions were performed by the two-sided Fisher's exact test (whenever 2x2 contingency tables are possible) or the χ^2 test (remaining cases). [a] $p < 0.05$ for the comparison of genotype distributions (dominant model) in the reference DTC population *versus* that in the study sample (70 mCi + 100 mCi groups combined). [b] $p < 0.05$ for the comparison of genotype distributions (codominant model) in the 70 mCi group *versus* that in the 100 mCi group. No further significant differences were found. MAF, minor allele frequency.

Table S2 – BNMN frequency (‰, mean ± S.D.) in DTC patients before and after (1, 3/6 and 24 months) therapy with different doses of ^{131}I (70 and 100 mCi).

	n [a]	t_0	t_1	t_3	t_6	t_{24}
**70 mCi**	15	5.27 ± 3.63	**8.80 ± 4.65***	**--**	**8.93 ± 5.92***	**9.64 ±2.80****
**100 mCi**	11	**9.64 ± 4.78 ‡**	**17.27 ± 5.14* ‡ ‡**	**21.40 ± 5.66*** ‡ ‡**	**--**	**--**
**TOTAL**	26	7.12 ± 4.63	**12.38 ± 6.39****	**--**	**--**	**--**

[a] At t_3, in the 100 mCi group, data was available for 10 patients only, while at t_{24}, in the 70 mCi group, only 11 patients were considered as the remaining 4 were submitted to further treatment.

* $p < 0.05$, compared to t_0; ** $p < 0.01$, compared to t_0; *** $p < 0.001$, compared to t_0; p-value for MN frequency comparison between different time points determined by the paired sample t test (whenever a normal distribution could not be excluded through the Shapiro-Wilk test) or the Wilcoxon signed-rank test (remaining cases); ‡ $p < 0.05$, compared to 70 mCi; ‡ ‡ $p < 0.001$, compared to 70 mCi; p-value for MN frequency comparison between different dose groups determined by the independent sample t test (whenever a normal distribution could not be excluded through the Shapiro-Wilk test) or the Mann-Whitney U test for independent samples (remaining cases).

Table S3 – Frequency of micronucleated cells (‰BNMN, mean ± SD) in the 70 mCi dose group at t_0, t_1, t_6 and t_{24}, and corresponding variation, according to genotype.

**Genotype**	**n**	**‰BNMN (mean ± SD)**				**Δ ‰BNMN (mean ± SD)**		
		**t_0**	**t_1**	**t_6**	**t_{24}**	**Δt_1**	**Δt_6**	**Δt_{24}**
***MLH1* rs1799977**								
Ile/Ile	7	4.14 ± 3.29	**12.14 ± 3.58**	10.86 ± 7.11	9.20 ± 1.30	**8.00 ± 4.97**	6.71 ± 6.85	5.00 ± 3.39
Ile/Val+Val/Val	8	6.25 ± 3.85	**5.88 ± 3.36***	7.25 ± 4.46	10.00 ± 3.74	**-0.38 ± 3.70***	1.00 ± 4.90	3.50 ± 4.37
***MSH3* rs26279**								
Thr/Thr	10	5.50 ± 3.63	8.90 ± 3.81	9.90 ± 7.09	10.13 ± 1.64	3.40 ± 3.47	4.40 ± 7.46	4.63 ± 3.96
Thr/Ala+Ala/Ala	5	4.80 ± 4.03	8.60 ± 6.54	7.00 ± 1.58	8.33 ± 5.13	3.80 ± 9.96	2.20 ± 3.70	3.00 ± 4.00
***MSH4* rs5745325**								
Ala/Ala	11	5.18 ± 3.79	8.91 ± 5.07	9.09 ± 6.64	9.63 ± 3.34	3.73 ± 6.83	3.91 ± 7.05	4.13 ± 3.91
Ala/Thr+Thr/Thr	4	5.50 ± 3.70	8.50 ± 3.87	8.50 ± 4.04	9.67 ± 0.58	3.00 ± 3.56	3.00 ± 4.90	4.33 ± 4.51
***PMS1* rs5742933**								
G/G	10	6.00 ± 4.08	9.20 ± 4.89	8.20 ± 3.77	9.88 ± 3.31	3.20 ± 7.05	2.20 ± 4.92	3.50 ± 4.14
G/C+C/C	4	4.75 ± 0.50	9.00 ± 4.55	12.00 ± 9.93	9.00 ± 0.00	4.25 ± 4.27	7.25 ± 9.67	4.50 ± 0.71
***MSH6* rs1042821**								
Gly/Gly	10	4.20 ± 3.55	8.40 ± 4.74	6.80 ± 2.57	8.43 ± 2.23	4.20 ± 6.16	2.60 ± 4.14	4.43 ± 4.47
Gly/Glu+Glu/Glu	5	7.40 ± 3.05	9.60 ± 4.88	13.20 ± 8.59	11.75 ± 2.63	2.20 ± 6.14	5.80 ± 9.78	3.75 ± 2.99
***RAD51* rs1801321**								
T/T	4	6.50 ± 3.70	10.25 ± 4.11	12.25 ± 9.74	9.33 ± 0.58	3.75 ± 4.86	5.75 ± 11.30	2.33 ± 3.79
T/G+G/G	11	4.82 ± 3.68	8.27 ± 4.90	7.73 ± 3.82	9.75 ± 3.33	3.45 ± 6.59	2.91 ± 4.06	4.88 ± 3.87
***NBN* rs1805794**								

Glu/Glu	7	5.43 ± 4.61	10.00 ± 4.51	8.14 ± 4.56	9.86 ± 2.12	4.57 ± 5.80	2.71 ± 4.89	4.43 ± 3.99
Glu/Gln+Gln/Gln	8	5.13 ± 2.85	7.75 ± 4.80	9.63 ± 7.15	9.25 ± 4.11	2.63 ± 6.44	4.50 ± 7.71	3.75 ± 4.11
***XRCC3* rs861539**								
Thr/Thr	5	4.40 ± 2.41	9.20 ± 3.83	9.00 ± 5.61	11.00 ± 2.65	4.80 ± 4.66	4.60 ± 5.13	6.67 ± 2.52
Thr/Met+Met/Met	10	5.70 ± 4.17	8.60 ± 5.19	8.90 ± 6.37	9.13 ± 2.85	2.90 ± 6.72	3.20 ± 7.15	3.25 ± 3.96
***XRCC5* rs2440**								
T/T	5	3.60 ± 1.67	8.40 ± 3.65	7.00 ± 3.16	9.33 ± 0.58	4.80 ± 4.15	3.40 ± 3.36	6.00 ± 2.65
T/C+C/C	10	6.10 ± 4.12	9.00 ± 5.25	9.90 ± 6.86	9.75 ± 3.33	2.90 ± 6.87	3.80 ± 7.64	3.50 ± 4.14

* $p < 0.05$; *p*-value for variant allele carriers *versus* common allele homozygotes determined by the Student t test (whenever a normal distribution could not be excluded through the Shapiro-Wilk test) or the Mann-Whitney U test (remaining cases).

Table S4 – Frequency of micronucleated cells (‰BNMN, mean ± SD) in the 100 mCi dose group at t_0, t_1 and t_3, and corresponding variation, according to genotype.

Genotype	n	‰BNMN (mean ± SD)			Δ ‰BNMN (mean ± SD)	
		**t_0**	**t_1**	**t_3**	**Δt_1**	**Δt_3**
***MLH1* rs1799977**						
Ile/Ile	3	**5.33 ± 1.16**	**24.00 ± 3.46**	21.50 ± 7.78	**18.67 ± 3.06**	16.50 ± 6.36
Ile/Val+Val/Val	8	**11.25 ± 4.62***	**14.75 ± 2.77***	21.38 ± 5.71	**3.50 ± 4.57***	10.13 ± 5.28
***MSH3* rs26279**						
Thr/Thr	8	8.00 ± 2.73	16.88 ± 5.79	**19.00 ± 4.93**	8.88 ± 7.72	10.71 ± 5.41
Thr/Ala+Ala/Ala	3	14.00 ± 7.00	18.33 ± 3.51	**27.00 ± 2.00***	4.33 ± 10.12	13.00 ± 7.55
***MSH4* rs5745325**						
Ala/Ala	4	13.25 ± 5.68	13.75 ± 3.50	25.50 ± 4.73	**0.50 ± 3.11**	12.25 ± 5.32
Ala/Thr+Thr/Thr	7	7.57 ± 2.88	19.29 ± 4.99	18.67 ± 4.68	**11.71 ± 7.27***	10.83 ± 6.49
***PMS1* rs5742933**						
G/G	9	9.56 ± 5.13	17.89 ± 5.53	22.25 ± 5.87	8.33 ± 8.79	12.25 ± 5.34
G/C+C/C	2	10.00 ± 4.24	14.50 ± 0.71	18.00 ± 4.24	4.50 ± 4.95	8.00 ± 8.49
***MSH6* rs1042821**						
Gly/Gly	9	10.00 ± 5.17	16.89 ± 3.44	20.56 ± 5.29	6.89 ± 7.04	10.56 ± 5.43
Gly/Glu+Glu/Glu	2	8.00 ± 2.83	19.00 ± 12.73	29.00 ± --	11.00 ± 15.56	19.00 ± --
***RAD51* rs1801321**						
T/T	4	8.50 ± 1.29	14.75 ± 3.40	20.75 ± 6.02	6.25 ± 4.35	12.25 ± 5.38
T/G+G/G	7	10.29 ± 5.99	18.71 ± 5.62	21.83 ± 5.95	8.43 ± 9.98	10.83 ± 6.46
***NBN* rs1805794**						
Glu/Glu	8	9.00 ± 4.84	**19.13 ± 4.64**	19.57 ± 4.89	10.13 ± 8.10	10.14 ± 6.20
Glu/Gln+Gln/Gln	3	11.33 ± 5.13	**12.33 ± 2.52***	25.67 ± 5.77	1.00 ± 3.61	14.33 ± 4.04
***XRCC3* rs861539**						
Thr/Thr	5	12.60 ± 5.60	17.40 ± 3.13	22.20 ± 6.72	4.80 ± 7.86	9.60 ± 7.30
Thr/Met+Met/Met	6	7.17 ± 2.14	17.17 ± 6.71	20.60 ± 5.03	10.00 ± 8.32	13.20 ± 3.70
***XRCC5* rs2440**						
T/T	2	7.50 ± 2.12	19.00 ± 4.24	22.50 ± 6.36	11.50 ± 6.36	15.00 ± 8.49
T/C+C/C	7	9.14 ± 4.53	16.57 ± 6.27	21.50 ± 6.19	7.43 ± 9.27	11.83 ± 5.53

* $p < 0.05$; *p*-value for variant allele carriers *versus* common allele homozygotes determined by the Student t test (whenever a normal distribution could not be excluded through the Shapiro-Wilk test) or the Mann-Whitney U test (remaining cases).

Table S5 – Frequency of micronucleated cells (‰BNMN, mean ± SD) in the combined dose groups at t_0 and t_1, and corresponding variation, according to genotype.

**Genotype**	**n**	**‰BNMN (mean ± SD)**		**Δ ‰BNMN (mean ± SD)**
		**t_0**	**t_1**	**Δt_1**
***MLH1* rs1799977**				
Ile/Ile	10	**4.50 ± 2.80**	**15.70 ± 6.63**	**11.20 ± 6.71**
Ile/Val+Val/Val	16	**8.75 ± 4.85***	**10.31 ± 5.46***	**1.56 ± 4.49***
***MSH3* rs26279**				
Thr/Thr	18	6.61 ± 3.42	12.44 ± 6.18	5.83 ± 6.22
Thr/Ala+Ala/Ala	8	8.25 ± 6.78	12.25 ± 7.31	4.00 ± 9.27
***MSH4* rs5745325**				
Ala/Ala	15	7.33 ± 5.55	**10.20 ± 5.09**	**2.87 ± 6.13**
Ala/Thr+Thr/Thr	11	6.82 ± 3.19	**15.36 ± 7.00***	**8.55 ± 7.41***
***PMS1* rs5742933**				
G/G	19	7.68 ± 4.83	13.32 ± 6.74	5.63 ± 8.13
G/C+C/C	6	6.50 ± 3.33	10.83 ± 4.54	4.33 ± 3.98
***MSH6* rs1042821**				
Gly/Gly	19	6.95 ± 5.20	12.42 ± 5.96	5.47 ± 6.55
Gly/Glu+Glu/Glu	7	7.57 ± 2.76	12.29 ± 7.99	4.71 ± 9.16
***RAD51* rs1801321**				
T/T	8	7.50 ± 2.78	12.50 ± 4.24	5.00 ± 4.47
T/G+G/G	18	6.94 ± 5.31	12.33 ± 7.26	5.39 ± 8.18
***NBN* rs1805794**				
Glu/Glu	15	7.33 ± 4.92	**14.87 ± 6.46**	7.53 ± 7.44
Glu/Gln+Gln/Gln	11	6.82 ± 4.40	**9.00 ± 4.69***	2.18 ± 5.67
***XRCC3* rs861539**				
Thr/Thr	10	8.50 ± 5.93	13.30 ± 5.44	4.80 ± 6.09
Thr/Met+Met/Met	16	6.25 ± 3.53	11.81 ± 7.04	5.56 ± 7.92
***XRCC5* rs2440**				
T/T	7	4.71 ± 2.50	11.43 ± 6.21	6.71 ± 5.38
T/C+C/C	17	7.35 ± 4.43	12.12 ± 6.71	4.76 ± 8.00

* $p < 0.05$; p-value for variant allele carriers *versus* common allele homozygotes determined by the Student t test (whenever a normal distribution could not be excluded through the Shapiro-Wilk test) or the Mann-Whitney U test (remaining cases).

Table S6 – Cytokinesis-Block Proliferation Index (CBPI, mean ± SD) in the 70 mCi dose group at t_0, t_1, t_6 and t_{24}, and corresponding variation, according to genotype.

Genotype	n	CBPI (mean ± SD)				ΔCBPI (mean ± SD)		
		t_0	t_1	t_6	t_{24}	Δt_1	Δt_6	Δt_{24}
***MLH1* rs1799977**								
Ile/Ile	7	1.73 ± 0.13	1.79 ± 0.20	1.69 ± 0.10	1.51 ± 0.12	0.06 ± 0.10	-0.04 ± 0.14	-0.23 ± 0.19
Ile/Val+Val/Val	8	1.82 ± 0.12	1.86 ± 0.13	1.78 ± 0.11	1.56 ± 0.07	0.05 ± 0.15	-0.03 ± 0.17	-0.25 ± 0.16
***MSH3* rs26279**								
Thr/Thr	10	1.76 ± 0.15	1.78 ± 0.16	1.74 ± 0.12	1.51 ± 0.10	0.01 ± 0.11	-0.02 ± 0.16	-0.25 ± 0.19
Thr/Ala+Ala/Ala	5	1.80 ± 0.07	1.94 ± 0.12	1.74 ±0.11	1.59 ± 0.02	0.14 ± 0.11	-0.06 ± 0.15	-0.24 ± 0.10
***MSH4* rs5745325**								
Ala/Ala	11	1.77 ± 0.14	1.81 ± 0.17	1.75 ± 0.11	1.56 ± 0.06	0.04 ± 0.13	-0.02 ± 0.16	-0.23 ± 0.17
Ala/Thr+Thr/Thr	4	1.79 ±0.11	1.89 ± 0.14	1.72 ± 0.11	1.48 ± 0.15	0.10 ± 0.08	-0.08 ± 0.13	-0.28 ± 0.18
***PMS1* rs5742933**								
G/G	10	1.76 ± 0.14	1.83 ± 0.17	1.76 ± 0.11	1.51 ± 0.09	0.06 ± 0.11	-0.01 ± 0.17	-0.24 ± 0.16
G/C+C/C	4	1.76 ± 0.07	1.83 ± 0.20	1.69 ± 0.12	1.65 ± 0.00	0.07 ± 0.17	-0.07 ± 0.05	-0.14 ± 0.06
***MSH6* rs1042821**								
Gly/Gly	10	1.79 ± 0.14	1.87 ± 0.17	1.73 ± 0.10	1.53 ± 0.08	0.09 ± 0.11	-0.06 ± 0.18	-0.25 ± 0.20
Gly/Glu+Glu/Glu	5	1.75 ± 0.11	1.75 ± 0.14	1.76 ± 0.13	1.54 ± 0.13	-0.01 ± 0.14	0.00 ± 0.06	-0.24 ± 0.11
***RAD51* rs1801321**								
T/T	4	1.70 ± 0.11	1.74 ± 0.16	1.72 ± 0.14	1.60 ± 0.09	0.04 ± 0.13	0.02 ± 0.17	-0.12 ± 0.05
T/G+G/G	11	1.80 ± 0.13	1.86 ± 0.16	1.75 ± 0.11	1.51 ± 0.09	0.06 ± 0.13	-0.06 ± 0.15	-0.29 ± 0.17
***NBN* rs1805794**								

Glu/Glu	7	1.76 ± 0.18	1.77 ± 0.17	1.78 ± 0.09	1.52 ± 0.09	0.00 ± 0.11	0.02 ± 0.16	-0.25 ± 0.18
Glu/Gln+Gln/Gln	8	1.79 ± 0.08	1.88 ± 0.15	1.71 ± 0.12	1.57 ± 0.10	0.10 ± 0.12	-0.08 ± 0.13	-0.24 ± 0.16
***XRCC3* rs861539**								
Thr/Thr	5	1.83 ± 0.08	1.89 ± 0.11	1.78 ± 0.13	1.53 ± 0.12	0.06 ± 0.13	-0.04 ± 0.14	-0.31 ± 0.19
Thr/Met+Met/Met	10	1.75 ± 0.15	1.80 ± 0.19	1.72 ± 0.10	1.54 ± 0.09	0.05 ± 0.13	-0.03 ± 0.16	-0.22 ± 0.16
***XRCC5* rs2440**								
T/T	5	1.80 ± 0.06	1.89 ± 0.10	1.74 ± 0.10	1.57 ± 0.13	0.10 ± 0.12	-0.06 ± 0.14	-0.25 ± 0.19
T/C+C/C	10	1.77 ± 0.16	1.80 ± 0.19	1.74 ± 0.12	1.52 ± 0.08	0.03 ± 0.13	-0.03 ± 0.16	-0.24 ± 0.17

p-value for variant allele carriers *versus* common allele homozygotes determined by the Student t test (whenever a normal distribution could not be excluded through the Shapiro-Wilk test) or the Mann-Whitney U test (remaining cases).
